# CHOP‐Mediated Disruption of Hippocampal Synaptic Plasticity and Neuronal Activity Contributes to Chronic Pain‐Related Cognitive Deficits

**DOI:** 10.1111/cns.70160

**Published:** 2025-01-16

**Authors:** Qingsheng Meng, Songxue Su, Lei Lei, Yubing Zhang, Jiabin Duan, Xiuhua Ren, Yihang Song, Xiaoyu Hu, Shiyue Chen, Weidong Zang, Zhen Zhang, Jing Cao

**Affiliations:** ^1^ Department of Anatomy, School of Basic Medical Sciences Zhengzhou University Zhengzhou Henan China; ^2^ Department of Anesthesiology, Pain and Perioperative Medicine The First Affiliated Hospital of Zhengzhou University Zhengzhou Henan China; ^3^ Neuroscience Research Institute Zhengzhou University Academy of Medical Sciences Zhengzhou Henan China; ^4^ Department of Anesthesiology The Affiliated Cancer Hospital of Zhengzhou University & Henan Cancer Hospital Zhengzhou Henan China

**Keywords:** CHOP, chronic pain, cognitive impairment, neuronal activity, synaptic plasticity

## Abstract

**Objectives:**

Endoplasmic reticulum (ER) stress‐induced protein homeostasis perturbation is a core pathological element in the pathogenesis of neurodegenerative diseases. This study aims to clarify the unique role played by C/EBP homologous protein (CHOP) as a biomarker of the unfolded protein response (UPR) in the etiology of chronic pain and related cognitive impairments following chronic constrictive nerve injury (CCI).

**Methods:**

The memory capability following CCI was assessed utilizing the Morris water maze (MWM) and fear conditioning test (FCT). Activation of the UPR was quantified by assessing levels of CHOP and key ER stress sensors. The terminal deoxynucleotidyl transferase (TdT) dUTP nick‐end labeling (TUNEL) assay and the levels of cleaved caspase‐3 were utilized to assess apoptosis level. Synaptic plasticity was assessed via a modified Golgi‐Cox staining method, and long‐term potentiation (LTP) measurements were taken. Neuronal activity was determined by immunofluorescence and fiber photometry. Knockdown of CHOP and alleviation of ER stress were selectively induced by LV‐Ddit3‐shRNAs and the chemical chaperone 4‐phenylbutyric acid (4‐PBA), respectively.

**Results:**

Mice subjected to CCI displayed enduring pain and cognitive impairments evident on Days 21–28 post‐surgery. Following CCI, changes in the dorsal CA1 (dCA1) manifested as ER dilation, upregulation of CHOP and upstream signaling molecules, reduced dendritic spine density, and PSD95 levels, and impaired LTP. Additionally, the co‐localization of CaMKIIα/c‐Fos and CaMKIIα^dCA1^‐mediated calcium signaling was significantly reduced, while the activation of CaMKIIα was found to mitigate cognitive impairments in CCI mice. Selective knockdown of CHOP enhanced synaptic plasticity and CaMKIIα neuron activity, while 4‐PBA treatment alleviated ER stress, synergistically improving cognitive deficits associated with chronic pain.

**Conclusion:**

CCI‐induced CHOP upregulation impairs dCA1 synaptic plasticity and neuronal activity, leading to chronic pain‐related cognitive deficits.

## Graphical abstract

Peripheral nerve injury triggers the UPR, increasing CHOP levels. Elevated CHOP inhibits synaptic plasticity and CaMKIIα^dCA1^ activity in dCA1 neurons, leading to cognitive deficits in chronic pain.

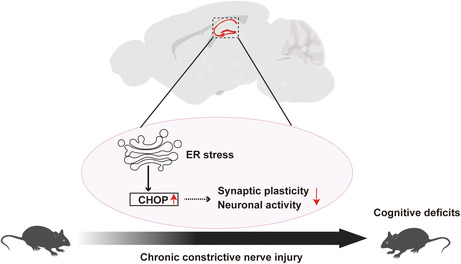

**Abbreviations**
4‐PBA: 4‐phenylbutyric acid
ACC: anterior cingulate cortex
ATF6: activating transcription factor 6
CCI: chronic constriction injury
CHOP: C/EBP homologous protein
FCT: fear conditioning test
fEPSP: field excitatory postsynaptic potential
Hip: hippocampus
IRE1: inositol‐requiring kinase 1
LTP: long‐term potentiation
mPFC: medial prefrontal cortex
OFT: open field test
PERK: protein kinase R‐like endoplasmic reticulum kinase
PSD95: postsynaptic density protein 95
PWF: paw withdrawal frequency
PWL: paw withdrawal latency
sh‐CHOP: shRNA‐CHOP (Ddit3)‐Mus‐414
sh‐NC: shRNA‐NC
TBS: theta burst stimulation
UPR: unfolded protein response

## 1 Introduction

Chronic pain represents a prevalent and intricate issue in global health, emerging as a cardinal factor leading to prolonged disability and a reduction in the overall quality of life across the world [1]. Epidemiological studies have shown that more than 50% of individuals with chronic pain experience cognitive decline, marked by impairments in attention, learning, memory, psychomotor skills, and executive functions [2, 3]. Corroborating these findings, preclinical research has demonstrated that rodents subjected to chronic pain display significant cognitive deficits [4]. Pathogenesis is multifactorial, involving plasticity alterations, neuroinflammation, neurotransmitter dysregulation, brain morphological and functional changes, epigenetics, nerve damage, stress, and medication effects [5]. Despite these insights, a comprehensive understanding of the mechanisms linking chronic pain to cognitive dysfunction remains elusive.

Chronic stress impairs neural plasticity and the equilibrium between excitability and inhibition, namely the E‐I balance, leading to various neurological disorders [6]. Extended exposure to pain stimuli induces substantial modifications in hippocampal neural plasticity, entailing enduring alterations in synaptic structure and function, thereby playing a role in the onset and perpetuation of diverse chronic pain conditions and their associated comorbidities [7]. Furthermore, maintaining the E‐I balance is crucial for proper neural functioning. The presence of persistent noxious stimuli during chronic pain can disturb this balance by modifying the mPFC and ACC neuronal activity [8, 9]. The ongoing stress‐induced aggregation and misfolding of neuronal proteins serve as key factors in the pathogenesis of synaptic dysfunction and changes in neuronal activity [10]. Consequently, investigating changes in protein homeostasis within the hippocampus, mPFC, and ACC warrants further research to deepen our understanding of these mechanisms.

The endoplasmic reticulum (ER) stress plays a pivotal role in maintaining protein homeostasis [11]. In response to stress, transient ER stress initiates adaptive mechanisms to restore protein balance, while persistent stimuli result in a detrimental ER stress‐induced UPR, causing disruption to proteostasis [11]. CHOP serves as a key effector in the UPR [12]. As a member of the C/EBP family, CHOP features an N‐terminal activation domain and a C‐terminal bZIP domain, with Ser79 and Ser82 crucial for kinase‐mediated regulation and intracellular activity [12]. While CHOP expression is typically low in the cytoplasm under physiological conditions, it is upregulated during periods of stress [12]. Upregulation of CHOP has the potential to initiate a cascade of cellular responses that could lead to protein homeostasis imbalance, compromising neuronal structure and function, ultimately resulting in apoptosis [13]. Research exploring the role of CHOP in cognitive impairments associated with chronic pain remains limited.

By constructing a neuropathic pain mouse model, this study aims to explore the impact of peripheral nerve injury on ER stress in brain areas intricately linked to pain, with a specific focus on the role of CHOP in chronic pain and its associated cognitive impairments.

## 2 Materials and Methods

### 2.1 Animals

All practices involving animal care and treatment used in this study were approved by the Animal Center of Henan Province in accordance with the guidelines issued by the Animal Care Committees of Zhengzhou University and the Life Sciences Ethics Review Committee of Zhengzhou University. Adult male C57BL/6 mice (8–12 weeks, 20–25 g) were purchased from the Laboratory Animals Center at Zhengzhou University. All mice were housed 3–5 per cage under typical laboratory conditions (22°C–25°C, 40%–60% humidity, 12‐h light or dark cycle, and with free access to food and water) and were allowed 7–10 days to acclimate to their surroundings before the beginning of experiments.

### 2.2 CCI‐Induced Neuropathic Pain Model

The CCI model operation was performed as previously reported [14]. In brief, mice were anesthetized with isoflurane (3% for induction and 1%–2% for maintenance, RWD Life Science Co, Shenzhen, China). An incision was made on the skin of the left midthigh to expose the sciatic nerve. Four ligatures were loosely tied around the sciatic nerve at 1 mm intervals with 5‐0 chromic gut (Boda Medical Products Co, Heze, China). The sham group mice underwent exposure of the sciatic nerve without ligation. The muscle and skin were sutured in layers with 4‐0 silk suture.

### 2.3 Behavioral Tests

All mice were acclimatized to the laboratory environment for 30 min per day for 2–3 days before behavior testing. Paw withdrawal frequency (PWF) and paw withdrawal latency (PWL) were measured before surgery and 7, 14, 21 and 28 days after CCI, respectively, to reflect the sensitivity to mechanical and thermal stimulation. The open field test (OFT) was performed to assess locomotion activity on Days 7 and 21 after surgery. For the assessment of learning and memory, we conducted the classical MWM and FCT on Days 7–12 and 21–28 after surgery. Throughout the study, all behavioral tests were carried out by an investigator blinded to the treatment group.

### 2.4 The PWF

Mice were placed on a metal mesh grid covered with a clear plastic chamber, and the calibrated von Frey filament (Muromachi Kikai, Tokyo, Japan) with a bending force of 0.07 and 0.4 g was applied ten times to the mid‐plantar surface of the hind paw. Each application was held for 3 s, and withdrawal, shaking, and licking were considered as positive responses. The number of withdrawal responses out of 10 stimulations was expressed as an overall percentage withdrawal frequency [(number of paw withdrawals/10 trials) × 100%].

### 2.5 The PWL

The mice were placed on a heated metal plate covered with an organic plastic box. Radiant heat stimulating the middle plantar surface of the hind paw was delivered through the glass plate (Ugo Basile S.R.L, Milan, Italy). The time(s) until the mice manifested a nociceptive behavior (such as licking, shaking, or raising the hind paw) was considered as the PWL. In order to avoid tissue damage, the heat source was set to a maximum of 20 s and would automatically cut off even if the mice did not lift their foot. Each experiment was repeated three times with a 5‐min interval, and the final score is presented as an average of the 3 obtained scores.

### 2.6 The OFT

General locomotion activity was evaluated by measuring the total distance traveled during the 5‐min OFT. The mice were placed in the center of a 50 × 50 × 50 cm^3^ Plexiglas box and their freely moving behavior was recorded for 5 min using the Video Tracking System of Smart v3.0 software (Panlab Harvard Apparatus, USA). After each test, the open field arena was thoroughly cleaned with 70% ethanol and dried before the next mouse was placed inside.

### 2.7 The MWM

The MWM was carried out on Days 7–12 and 21–26 post‐surgery, respectively. The Morris water maze, equipped with a ceiling‐mounted camera and Video Tracking System software (XinRuan Co, Shanghai, China), is a circular tank with a diameter of 120 cm and a depth of 50 cm. Before testing, the water in the tank was colored with a white non‐toxic dye and maintained at a temperature of 23°C–25°C. A clear plexiglass goal platform, 9 cm in diameter, was submerged 0.5 cm below the water's surface in the southwest quadrant. Distinct graphic cues in four orientations were set to facilitate spatial orientation and the acquisition of spatial memory. The Video Tracking System software started recording the moment the mouse was released and stopped the trial once the animal had reached the hidden platform or the current training time (60 s) was over. During four trials on each of the five consecutive days, the animals were released facing the tank wall at one of four positions and allowed to swim freely within 60 s to find the hidden platform. If they did not find it, they were guided to the platform by the experimenter and remained on it for 10 s. On Day 6 of the probe trial, the platform was removed in advance. The animals were released in the opposite quadrant of the target quadrant, and the parameters involving the latency to the platform and the percentage of time spent in the target quadrant within 60 s were recorded.

### 2.8 The FCT

The FCT was conducted on Days 26–28 post‐surgery. The conditioned fear apparatus consists of a 30 × 30 × 45 cm^3^ fear box and a controller. The fear box features an electric fence on its bottom layer and is housed within a soundproof box. On the 26th day after surgery, the mice adapted to their cage environment. The next day, the mice were put into the test box for 180 s and then given sound stimulation (90 dB, 3000 Hz) for 20 s. At the end of the sound stimulation, a plantar electric shock (0.2 mA) was given for 2 s. The cycle lasted for 180 s, and the mice were put into the feeding cage after 4 cycles. Twenty‐four hours after the end of training, the mice were put back into the original test box without any stimulus, and the freezing time and number of times within five minutes were recorded. Two hours after the contextual conditioning test, the cued and fear condition test was carried out. The mice were placed in a test box with a changed environment (the four walls of the test box were covered with striped plastic plates), first adapted for 180 s, and then given 20 s of sound (90 dB, 3000 Hz). The cycle lasted for 180 s for 3 cycles. The duration and frequency of mouse rigidity were recorded. The entire experiment process was recorded by Shanghai Xinsoft's automatic monitoring system (XinRuan Co, Shanghai, China).

### 2.9 Golgi Staining

Golgi staining was performed using the FD Rapid GolgiStain Kit (FD Neuro Technologies, USA). The brains obtained from mice were immersed in the impregnation solution prepared by mixing equal volumes of Solutions A and B and kept in the dark at room temperature for 14 days. The impregnation solution was replaced in the first 24 h. Then, the brain tissues were transferred to Solution C, which was replaced again after 24 h, and stored at 4°C in the dark for 5 days. The brains were sliced with a vibrating microtome (Leica Microsystems, Germany) into sections of 100 μm thickness, and the sections were mounted on gelatin‐coated microscope slides with Solution C. The sections were rinsed twice for 4 min each in distilled water, and then placed in a 1:1:2 mixture of Solution D, Solution E, and Milli‐Q water for 10 min. After rinsing twice in distilled water, the sections were dehydrated in ethanol with graded concentrations (50%, 75%, 95%) for 4 min each, and finally in 100% ethanol for 4 times for 4 min each. The sections were then cleared and rinsed 3 times for 4 min each in xylene, and then cover‐slipped with Permount solution. The images were captured using confocal microscopy (Nikon, Tokyo, Japan). Sholl analysis was used to quantify the dendritic branches and overall dendritic length.

### 2.10 LTP Recording

Mice were anesthetized with 2% isoflurane and decapitated, their brains were quickly removed and chilled in ice‐cold sucrose‐based artificial cerebrospinal fluid (ACSF) saturated with 95% O_2_ and 5% CO_2_ (composition in mM: 130 NaCl, 3.5 KCl, 1.25 NaH_2_PO_4_, 1.5 MgSO_4_·7H_2_O, 2.0 CaCl_2_, 24 NaHCO_3_, 10 glucose, pH 7.2–7.4). Transverse hippocampal slices (300 μm thickness) were prepared using a vibrating microtome (Leica Microsystems, Germany). Slices were allowed to recover for 30 min at 32°C. Then, they were preincubated for at least 1 h at room temperature in a modified ACSF containing the following (composition in mM: 124 NaCl, 26 NaHCO_3_, 2.5 KCl, 1.25 NaH_2_PO_4_, 4 CaCl_2_, 4 MgCl_2_, and 20 glucose, saturated with 95% O_2_ and 5% CO_2_, pH 7.2–7.4). The Schaffer collateral branches were stimulated with a bipolar metal stimulating electrode, and the LTP of the CA1 area was recorded in current‐clamp mode using a glass microtubule pipette (1–3 MOmega) filled with ACSF. Five episodes of theta‐burst stimulation (TBS) with 10‐s intervals were used to induce LTP. Each episode contained 5 bursts at 5 Hz, with each burst consisting of 5 pulses at 100 Hz. Before LTP induction, a stable baseline fEPSP was recorded for 20 min. The magnitude of LTP was calculated by averaging fEPSP slopes from 50 to 60 min after the high‐frequency stimulation.

### 2.11 Western Blotting

Mice were terminally anesthetized with isoflurane, and the brain was removed and dissected into the mPFC, ACC, and hippocampus on ice. Immediately, RIPA lysis buffer (Beyotime, Shanghai, China) containing PMSF and a phosphatase inhibitor was used to homogenize the tissues. The concentration of proteins was measured with a BCA kit (Beyotime, Shanghai, China). The proteins, resuspended with loading buffer, were boiled for 5 min and kept at −80°C for Western blotting analysis. Twenty microgram protein samples were separated by 10% or 12% sodium dodecyl sulfate‐polyacrylamide gel electrophoresis (Bio‐Rad, Hercules, CA) for 40 min at 80 V, followed by 1–1.5 h at 120 V. After electrophoresis, the isolated proteins were transferred to polyvinylidene difluoride membranes (Millipore, Sunnyvale, USA). The membranes were then blocked with 5% bovine serum albumin in PBS containing 0.1% Tween 20 for 2 h at room temperature, followed by incubation with primary antibodies overnight at 4°C and then washed three times with TBST. The secondary antibodies conjugated to horseradish peroxidase (HRP) were incubated with the membranes for 2 h at room temperature and washed in TBST three times. Targeted protein bands were visualized with an ECL detection kit (Thermo Fisher Scientific, Rockford, USA), and band intensities were quantified using ImageJ software. The primary antibodies included anti‐PSD95 (1:1000, 2507S, Cell Signaling Technology), anti‐CHOP (1:1000, BF8018, Affinity), anti‐caspase 3 (1:5000, 82202‐1‐RR, Proteintech), anti‐cleaved caspase 3 (1:5000, 82707‐13‐RR, Proteintech), anti‐PERK (1:1000, BF8011, Affinity), anti‐p‐PERK (1:2000, 3192, Cell Signaling Technology), anti‐IRE1 (1:1000, DF7709, Affinity), anti‐p‐IRE1 (1:1000, AF7150, Affinity), anti‐ATF6 (1:1000, DF6009, Affinity), and anti‐Tubulin (1:5000, 11224‐1‐AP, Proteintech).

### 2.12 Quantitative Real‐Time Reverse Transcription PCR (qRT‐PCR)

Mice were humanely euthanized using isoflurane, followed by rapid decapitation. The hippocampus was promptly extracted from the animals. Total RNA was extracted using the Axyprep Total RNA Isolation Kit, and RNA concentration and purity were assessed spectrophotometrically at 260 and 280 nm using equipment from Thermo Fisher Scientific, USA. GAPDH served as the endogenous reference gene for normalization of CHOP mRNA levels. The Revert Aid First Strand cDNA Synthesis Kit (Thermo Fisher Scientific, USA) was utilized for the reverse transcription of mRNA into cDNA. Quantitative PCR was conducted on the iQ5 Real‐Time PCR Detection System (Bio‐Rad) with the SYBR Green qPCR Master Mix Kit (Thermo Fisher Scientific, USA). The PCR protocol was as follows: one cycle at 50°C for 2 min, one cycle at 95°C for 10 min, and 40 cycles at 95°C for 15 s and 60°C for 60 s. The relative expression of CHOP mRNA was quantified using the 2^−ΔΔCt^ method. The primer sequences utilized are detailed as follows: GAPDH (forward, 5′‐ATG GGT GTG AAC CAC GAG A‐3′ and reverse, 5′‐CAG GGA TGA TGT TCT GGG CA‐3′); Ddit3 (forward, 5′‐TCA CAC GCA CAT CCC AA‐3′ and reverse, 5′‐CCA CTC TGT TTC CGT TTC C‐3′).

### 2.13 Immunofluorescence

Upon completion of the cognitive behavioral testing within 2 h, animals were deeply anesthetized with sodium pentobarbital (60 mg/kg, i.p.) and then intracardially perfused with phosphate‐buffered saline (PBS) followed by fresh 4% paraformaldehyde. The brains were harvested, postfixed for 6–8 h at 4°C, and dehydrated in graded sucrose (20% and 30%) at 4°C until they sank. The brains were then embedded in an optimal cutting temperature (OCT) compound and frozen at −20°C. Frozen brains were sliced coronally into 30 μm sections containing the mPFC, ACC, and hippocampus using a Leica CM 3050S cryostat. After being rinsed in PBS three times, the brain sections were incubated in a blocking solution containing 10% normal donkey serum and 0.05% Triton X‐100 in PBS for 2 h at room temperature. They were then incubated at 4°C overnight with the following primary antibodies: mouse anti‐CHOP (1:200, 2895, Cell Signaling Technology), mouse anti‐c‐Fos (1:500, ab208942, Abcam), rabbit anti‐NeuN (1:200, 26,975‐1‐AP, Proteintech), rabbit anti‐GFAP (1:1000, ab7260, Abcam), rabbit anti‐Iba1 (1:1000, ab178846, Abcam), and rabbit anti‐CaMKIIα (1:200, ab103840, Abcam). The next day, the brain sections were washed three times for 10 min in 0.1% Triton‐X/PBS and then incubated with donkey anti‐rabbit or donkey anti‐mouse secondary antibodies conjugated with Alexa Fluor 488 or Alexa Fluor 555 (1:400, Jackson ImmunoResearch, USA). The nuclei were stained with DAPI (ab104139, Abcam). Finally, the images were captured using confocal microscopy (Nikon, Tokyo, Japan).

### 2.14 TUNEL Staining

Mice were infused with PBS and 4% PFA, and the brains were prepared into 3 μm sections. The sections were stained according to the instructions of the Cy3 TUNEL Apoptosis Detection Kit (AiFang, Hunan, China). After dewaxing the sections, antigen repair was performed at 37°C with a 20 μg/mL Proteinase K solution for 30 min. The brain slices were soaked in 0.5% triton X‐100/PBS for 20 min at room temperature to rupture the membranes. The TdT incubation buffer was added to the slices in accordance with the instructions and incubated at 37°C for 1 h. Each step was followed by a wash with PBS. Nuclear staining was performed with DAPI and the sections were sealed with an anti‐fluorescence quencher.

### 2.15 Transmission Electron Microscopy (TEM)

The hippocampal CA1 region was separated, immediately cut into 1 × 1 × 1 mm^3^ specimens, and fixed with 2.5% glutaraldehyde for 24 h at 4°C. After being washed with phosphate buffer, these samples were post‐fixed in 1% osmic acid for 2 h at 4°C and dehydrated with an acetone gradient. They were then embedded in epoxy at 37°C for 12 h and placed in an oven at 65°C for another 48 h. Slices, ranging in thickness from 50 to 70 nm, were mounted on grids, stained with uranyl acetate and lead citrate, and observed using a transmission electron microscope (Hitachi, Japan).

### 2.16 Virus Injection, Fiber Photometry, and Cannula Implantation

AAV2/9‐CaMKIIα‐GCaMP6s and AAV2/9‐CaMKIIα‐hM3D(Gq)‐mCherry, LV‐Ddit3‐shRNA were purchased from BrainVTA Co. Ltd. and GenePharma Co. Ltd., respectively. Mice were anesthetized with isoflurane and restrained in a stereotaxic apparatus (RWD Life Science, Shenzhen, China). Throughout all surgical and experimental procedures, a homeothermic heating blanket was set at 37°C to maintain the body temperature of the mice. A microsyringe injector and controller (RWD Life Science, Shenzhen, China) were used to inject the virus at a constant rate of 20–50 nL/min. After each injection, the needle was kept at the injection site for 10 min to prevent reflux. For chemogenetic manipulation, the AAV2/9‐CaMKIIα‐hM3D(Gq)‐mCherry or the control AAV2/9‐CaMKIIα‐mCherry was injected into the dorsal hippocampal CA1 area (bregma, −1.95 mm; midline, ±1.40 mm; skull surface, −1.4 mm). Following virus injection, 1 mg/kg clozapine‐N‐oxide (CNO) was injected intraperitoneally to observe the effect of neuronal activity on pain and chronic pain‐evoked memory and learning deficits behaviors.

For fiber photometry, after the deliberation of AAV2/9‐CaMKIIα‐GCaMP6s into the dorsal hippocampal CA1 area, the optic fiber was placed at the site (bregma, −1.95 mm; midline, ±1.40 mm; skull surface, −1.3 mm). A fiber photometry system (RWD Life Science, Shenzhen, China) was used to record the fluorescence signals generated by 470 nm LED light and 410 nm LED light excitation. After a 5‐min recording of basal fluorescence post‐acclimation, mice were exposed to sound stimulation and contextual conditioning test. DeltaF/F was calculated according to (470 nm signal‐fitted 410 nm signal)/(fitted 410 nm signal). The formula was as follows: *Z* score = (*x*‐mean)/std., *x* = delta F/F.

For cannula implantation, a guide cannula (RWD Life Science Co., Shenzhen, China) was implanted into the dorsal hippocampal CA1 area on the 7th day after CCI surgery. Catheters were placed for 1 week before administration to ensure proper healing.

After the experiments, coronal sections were collected to verify the locations of viral transduction, the cannula, and the optical fiber.

### 2.17 Statistical Analysis

Data in this study were presented as mean ± SEM and analyzed using GraphPad Prism 8.0 (GraphPad Inc., San Diego, CA, USA). Normality was assessed for all data before analysis using the Shapiro–Wilk test to test for normal distribution. Student's *t*‐test was used for two‐group comparisons with normally distributed data and uniform variance, while one‐way analysis of variance (ANOVA) was used for three or more groups. Two‐way repeated measures ANOVA was employed to compare two variables in two or more groups. Post hoc analysis using Tukey's multiple comparisons test was conducted following ANOVA. For non‐normally distributed data, the Mann–Whitney *U* test and Kruskal–Wallis test were applied. Statistical significance was set at *p* < 0.05.

## 3 Results

### 3.1 Chronic Sciatic Nerve Constrictive Injury Resulted in Nociceptive Hypersensitivity and Cognitive Deficits

**FIGURE 1:**
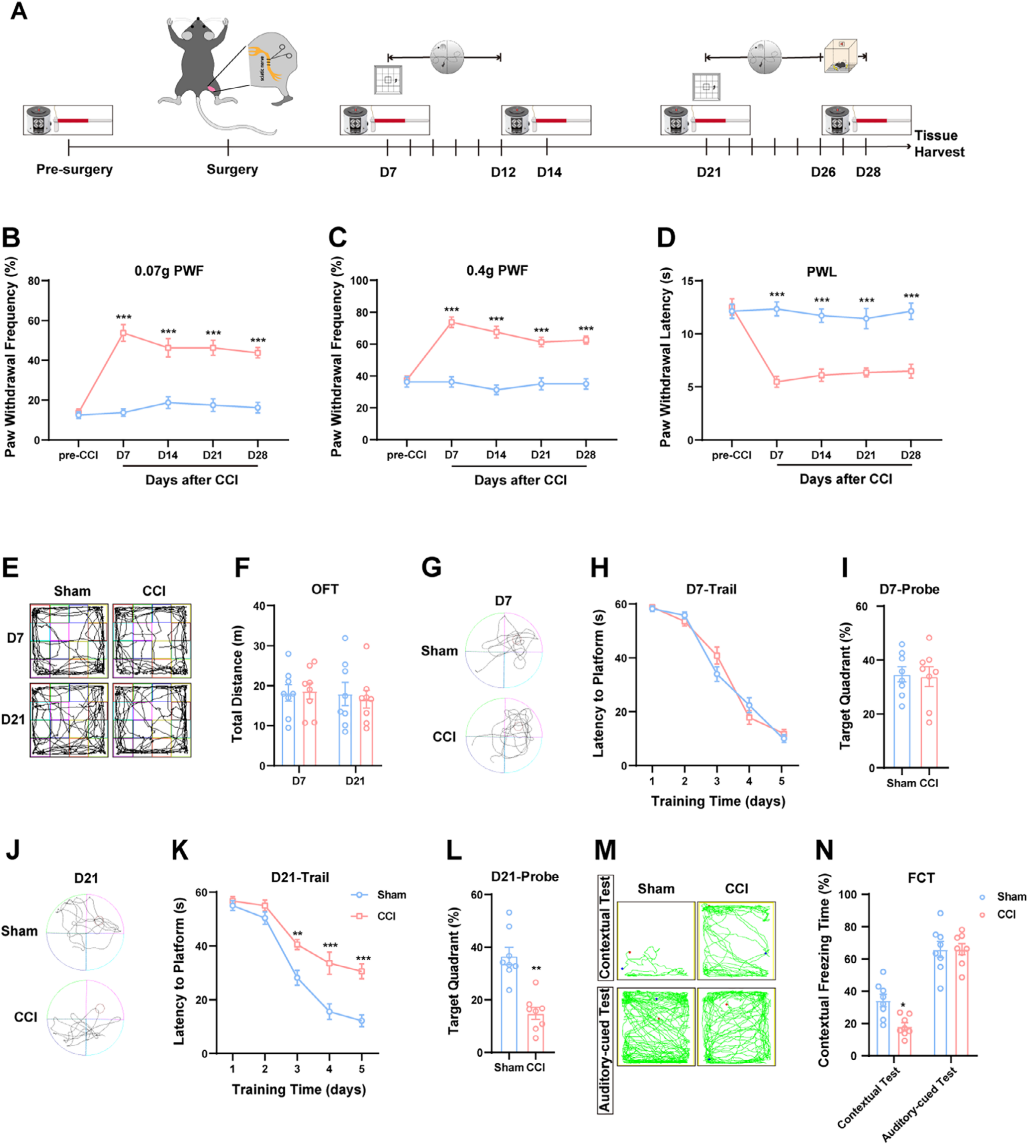
Chronic sciatic nerve constriction injury results in nociceptive hypersensitivity and cognitive deficits. (A) Schematic timeline of the experiment. (B, C) Time course of CCI‐induced mechanical hypersensitivity (paw withdrawal responses to 0.07 g and 0.4 g, respectively). (D) Time course of CCI‐induced thermal hyperalgesia. (E, F) The motor capacity manifested as total distance traveled was measured in OFT following CCI or sham surgery. (G–N) Learning and memory functionality was evaluated in MWM and FCT during the period spanning 21–28 days post‐surgery. *n* = 8 mice per group. *p < 0.05, **p < 0.01 and ***p < 0.001 as compared to sham mice.

Chronic pain induced by CCI has been extensively utilized and rigorously validated in research [15]. Figure 1A illustrates the experimental timeline for model establishment and behavioral assessments. As anticipated, PWF increased with both 0.07 and 0.4 g von Frey stimulation, while PWL decreased in response to heat stimulation in CCI mice at Days 7, 14, 21, and 28 post‐surgery, indicating that nerve damage resulted in significant mechanical allodynia and thermal hyperalgesia compared to the sham group (Figure 1B–D). Notably, there was no difference in responses to mechanical or thermal stimuli between sham‐operated and CCI mice prior to the CCI procedure (Figure 1B–D). Motor function was evaluated using OFT on Days 7 and 21 following surgery. As depicted in Figure 1E,F, the distance traveled by CCI mice did not differ from that of sham‐operated controls, suggesting that motor ability remained unaffected by the injury. Given the high prevalence of chronic pain associated with cognitive dysfunction, we subsequently assessed cognitive performance in CCI mice utilizing conventional MWM and FCT on Days 7 and 21 post‐surgery. Consistent with previous findings [7, 16], CCI mice exhibited a prolonged latency to locate a hidden platform as well as reduced occupancy within the target quadrant at Day 21–26 compared to controls. These results indicate impaired cognitive function and learning capabilities among CCI subjects (Figure 1J–L). However, no differences were observed between sham‐operated and CCI groups regarding learning and memory functions at Day 7–12 post‐surgery (Figure 1G–I), which suggests that cognitive dissonance arises during sustained pain rather than its initial development. In the FCT tone cue test, freezing behavior was comparable across both groups; however, during contextual testing, mice with CCI exhibited notably decreased freezing durations relative to their sham counterparts (Figure 1M,N). Collectively, these behavioral outcomes suggest that CCI can precipitate neuropathic pain alongside cognitive dysfunction.

### 3.2 Upregulated CHOP in the Hippocampus of CCI Mice Occur Without Apoptosis

**FIGURE 2:**
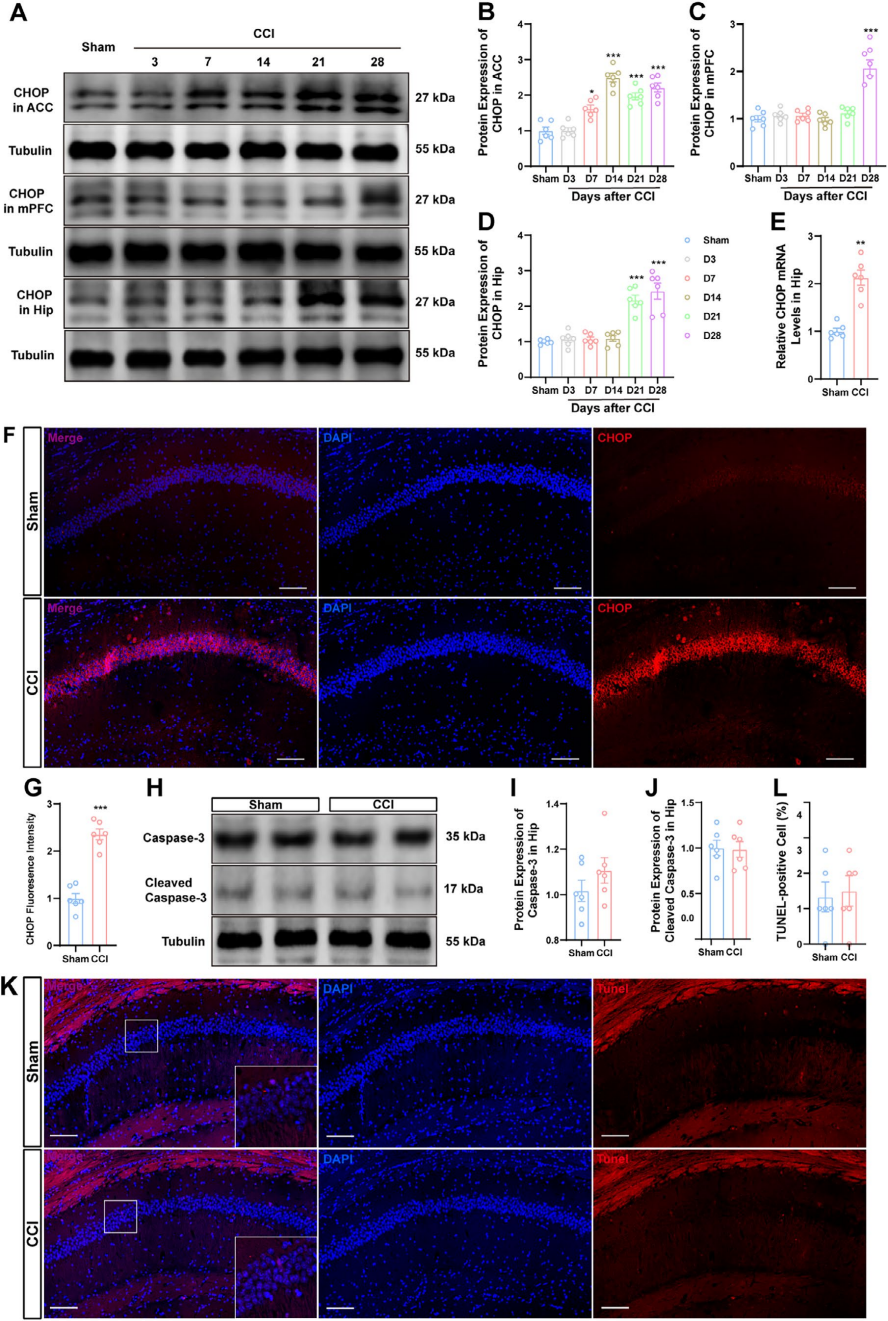
Upregulated CHOP in the hippocampus of CCI mice occur without apoptosis. (A–D) The expression patterns of CHOP in ACC, mPFC and hippocampus of CCI mice were determined by Western blotting respectively. (E) The mRNA expression of CHOP in the hippocampus following CCI was determined by RT‐qPCR. (F, G) Representative images of immunohistochemical staining for CHOP positive signals in the hippocampus of sham and CCI mice. Scale bar, 100 μm. (H–J) The protein expressions of cleaved caspase‐3 and total caspase‐3 were examined by Western blotting. (K, L) Representative images of immunohistochemical staining for TUNEL‐positive cells in the hippocampus of sham and CCI mice. Scale bar, 100 μm. *n* = 6 mice per group. *p < 0.05, **p < 0.01 and ***p < 0.001 as compared to sham mice.

Given the critical role of ER stress in the modulation of neurodegenerative diseases [10], we investigated the expression pattern of UPR marker CHOP across various regions, including the mPFC, ACC, and hippocampus following nerve injury. Western blotting analysis revealed a marked increase in CHOP expression within the ACC at 7, 14, 21, and 28 days post‐CCI, whereas mPFC exhibited enhanced CHOP levels only at 28 days after CCI (Figure 2A–C). In terms of hippocampal CHOP expression patterns, we noted elevations at both 21 and 28 days postoperatively. Notably, previous behavioral assessments indicated that hippocampal CHOP expression correlated with cognitive‐behavioral deficits (Figure 2A,D). Consequently, considering the timing of cognitive deficit onset, our follow‐up study focused on elucidating the role of CHOP in the hippocampus. We similarly observed a consistent increase in both hippocampal CHOP mRNA expression (Figure 2E) and fluorescence intensity in dCA1 (Figure 2F,G) at Day 28 post‐surgery. Considering that CHOP is pivotal to neuronal apoptosis pathophysiology [13], we subsequently examined cleaved caspase‐3 expression as a key effector of apoptotic processes [17]. As illustrated in Figure 2H–J, no significant differences were observed in the expression levels of full‐length caspase‐3 and cleaved caspase‐3 between the sham operation group and the CCI group, suggesting that apoptosis is not implicated under these conditions. TUNEL staining further corroborated this finding (Figure 2K,L). The findings suggest that increased CHOP in the hippocampus after CCI may not lead to significant apoptosis.

### 3.3 The Suppressed Synaptic Plasticity Following CCI

**FIGURE 3:**
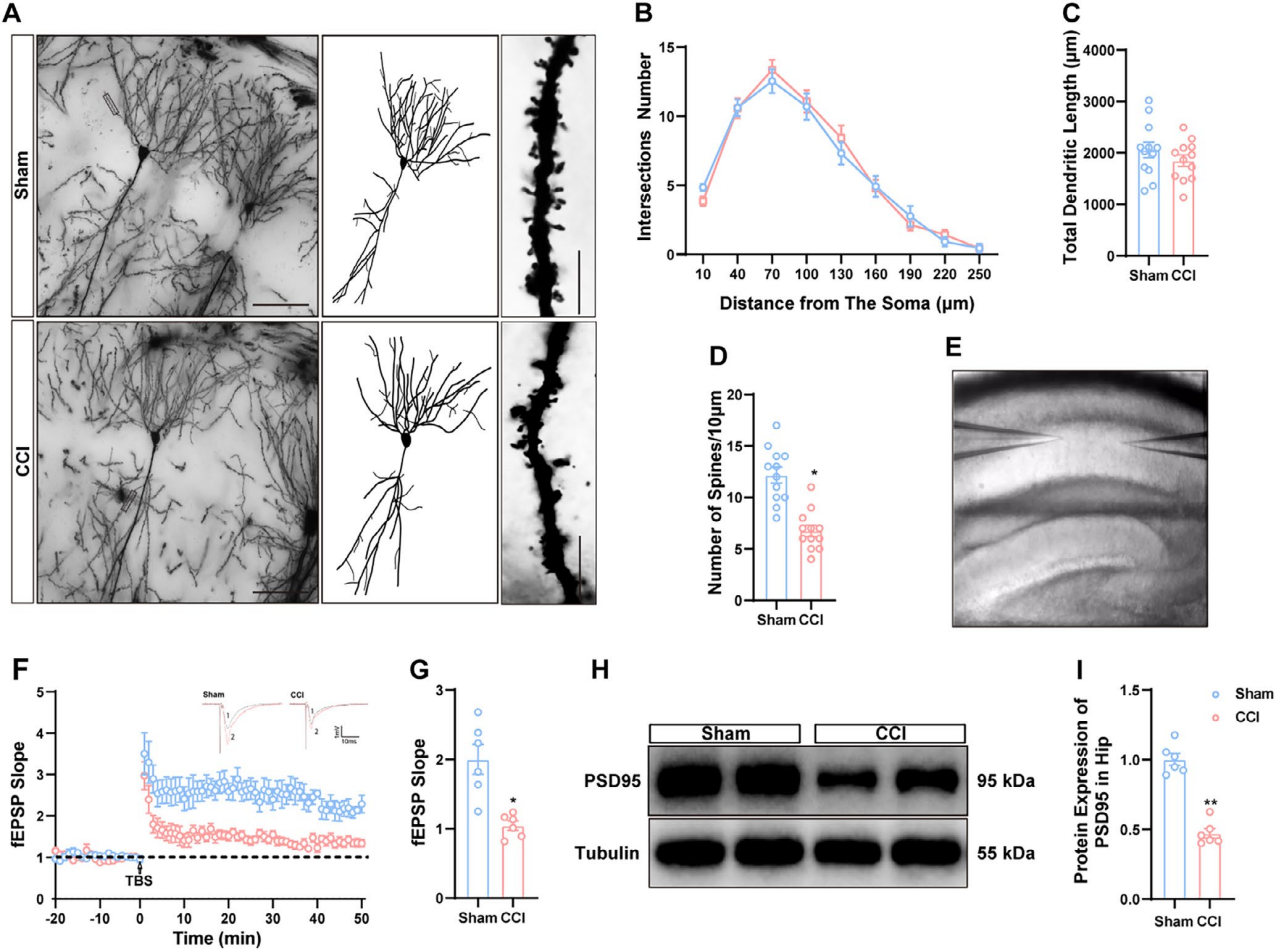
Chronic pain resulted in the impairment of synaptic plasticity in the hippocampus of CCI mice. (A–D) Presentation of images and quantitative analysis of Golgi staining to measure spine density and dendritic complexity in sham‐operated and CCI mice. Scale bar, 50 μm, 10 μm. (E) Representative images depicting the positions of recording and stimulating electrodes employed in LTP experiments. (F) LTP slope was recorded before and after TBS. (G) The average slope of LTP at 50–60 min following TBS. (H, I) Representative immunoblots and quantification of PSD95 expression in both groups. *n* = 6 mice per group. *p < 0.05, **p < 0.01, and ***p < 0.001 as compared to sham mice.

Golgi‐Cox staining was utilized to evaluate the effects of CCI on hippocampal neuronal dendrites and dendritic spines, as synaptic connections are essential for learning and memory [5]. No significant differences were detected in the total number of basal intersections or dendrite length between the sham operation group and the CCI group (Figure 3A–C). However, a significant reduction in dendritic spine density was observed in dCA1 pyramidal neurons within the CCI group, indicating altered synaptic plasticity during chronic pain (Figure 3D). Furthermore, we assessed LTP as it pertains to functional synaptic plasticity [7]. In alignment with Golgi‐Cox staining results, LTP slope significantly decreased following TBS in the CCI group, suggesting impaired synaptic plasticity within the hippocampal CA3‐CA1 circuit post‐CCI (Figure 3E–G). At a molecular level, we examined the expression of PSD95 as it interacts with various postsynaptic membrane proteins, participating in the regulation of synaptic morphology and function [18]. As illustrated in Figure 3H,I, the protein levels of PSD‐95 were significantly lower in the CCI group compared to controls. The results indicate that the synaptic structure and functional plasticity in the hippocampus of CCI mice are compromised.

### 3.4 Targeted CHOP Knockout Restored the Impairment of Synaptic Plasticity

**FIGURE 4:**
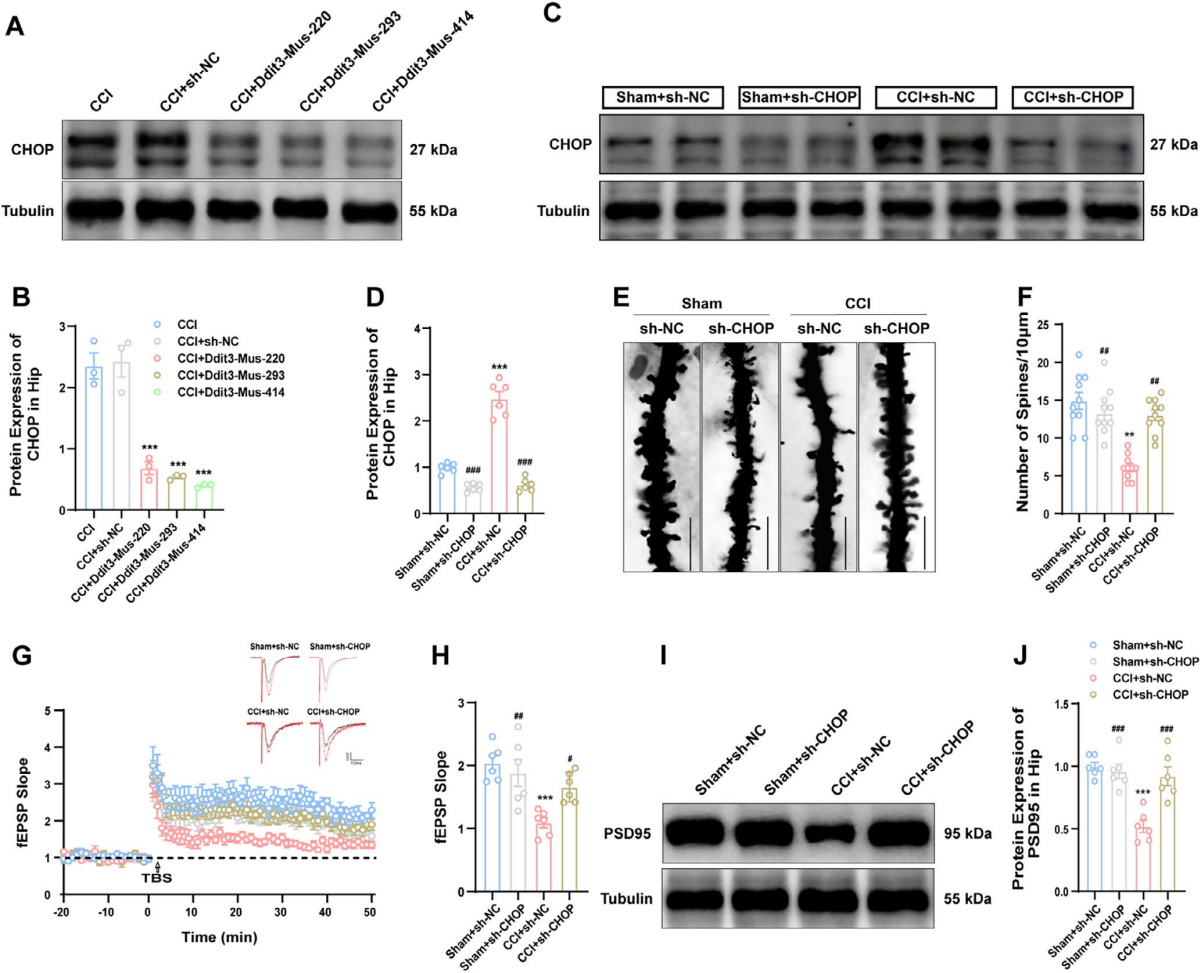
Downregulation of CHOP in CA1 improves the impaired synaptic plasticity in CCI mice. (A, B) The knockdown efficacy of three LV‐shRNA‐DDIT3 constructs on CHOP was determined by Western blotting. (C, D) The influence of LV‐shRNA‐DDIT3‐mus‐414 on the protein expression of CHOP. (E, F) Effect of CHOP knockdown on dendritic spine density in CA1 regions determined using Golgi staining. Scale bar, 10 μm. (G, H) Influence of CHOP knockdown on LTP slope. (I, J) Impact of CHOP downregulation on PSD95 protein expression. *n* = 6 mice per group. *p < 0.05, **p < 0.01, ***p < 0.001 versus with the sham + sh‐NC group; ^#^ *p* < 0.05, ^##^ *p* < 0.01, ^###^ *p* < 0.001 versus CCI + sh‐CHOP group.

To investigate whether the upregulation of CHOP expression is involved in synaptic plasticity impairment following CCI, we utilized shRNA‐DDIT3 (targeting CHOP) to downregulate its expression. Three viruses targeting CHOP were designed: LV‐DDIT3‐mus‐220, −293, and −440. These three shRNA‐DDIT3 (CHOP) or control viruses were injected into the dCA1 region of the hippocampus on Day 7 post‐surgery to assess the knockout efficiency. As depicted in Figure 4A,B, among these three viruses, LV‐DDIT3‐mus‐414 exhibited superior knockout efficacy and was employed for subsequent experiments. Similarly, elevated CHOP expression observed in the CCI + sh‐NC group was effectively inhibited by sh‐CHOP application (Figure 4C,D). Next, we investigated the effects of CHOP on synaptic plasticity by employing shRNA to achieve its knockdown. We observed that viral‐mediated downregulation of CHOP expression reversed the CCI‐induced reduction in dendritic spine density (Figure 4E,F) and restored the decreased slope of LTP induced by CCI (Figure 4G,H). Meanwhile, the decreased expression of PSD95 caused by CCI was successfully restored by inhibiting CHOP (Figure 4I,J). Targeting the downregulation of CHOP has the potential to reverse synaptic plasticity impairments observed after CCI, as indicated by these findings.

### 3.5 Chemogenetic Activation of CaMKIIα^dCA1^ Neurons Alleviated CCI‐Induced Memory Impairments, Without Influencing Pain Sensitivity

**FIGURE 5:**
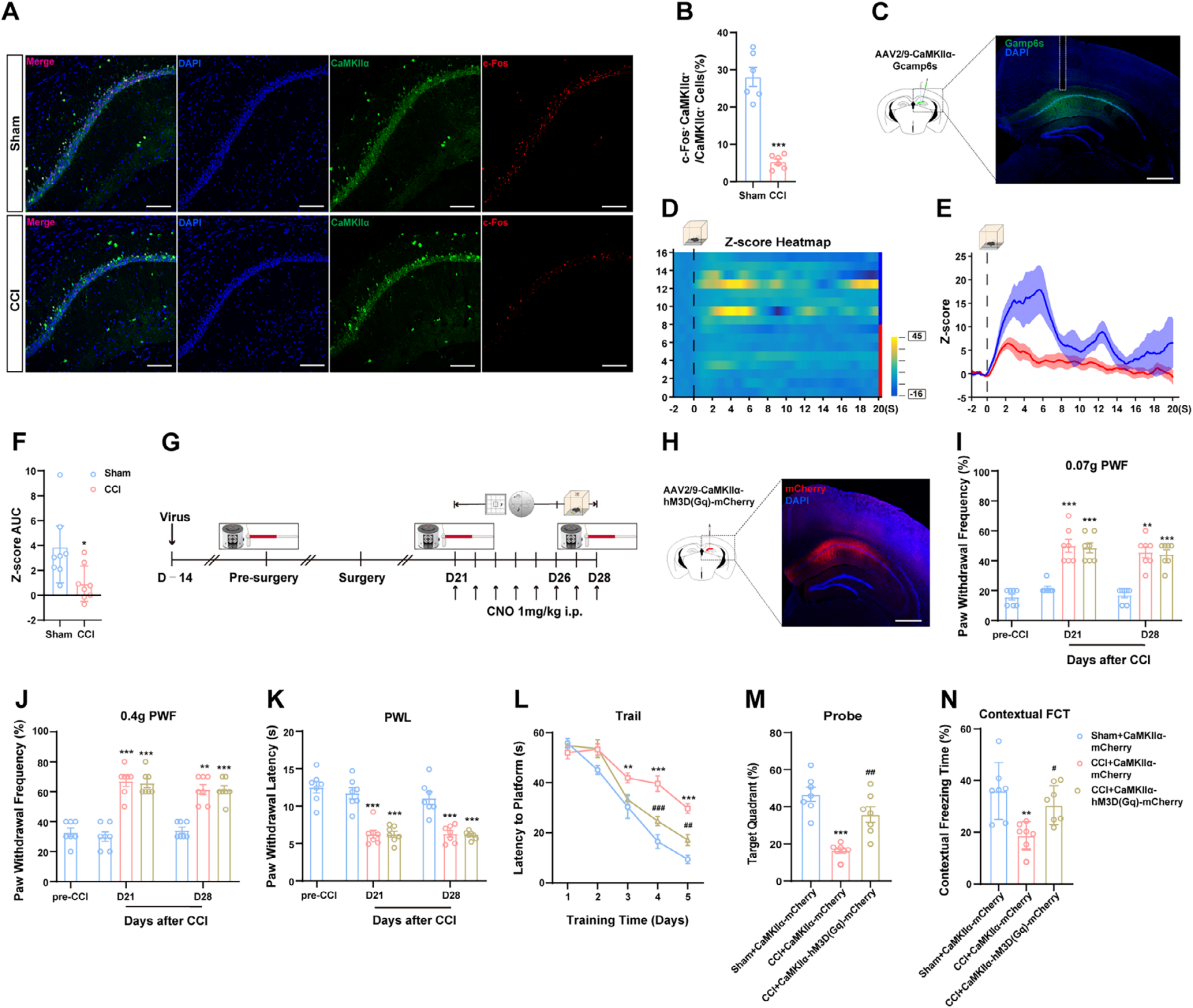
Activation of CaMKIIα^dCA1^ neurons ameliorates chronic pain‐related cognitive deficits in CCI mice. (A, B) Representative images of immunohistochemical staining for c‐Fos colocalization. Scale bar, 10 μm. (C) Schematic of AAV2/9‐CaMKIIα‐GCaMp6s‐WPRE injection and optical fiber implantation in CA1 region. Scale bar, 200 μm. (D–F) Representative images of Z‐score heatmap and Z‐score AUC for Calcium signaling during the contextual FCT in CCI mice. (G) Timeline of the experiments for chemogenetic viral injection and behavioral tests. (H) Diagram showing the injection site of AAV2/9‐CaMKIIα‐hM3D(Gq)‐mCherry in the CA1 region. Scale bar, 200 μm. (I–K) Effect of chemogenetic activation of CA1 CaMKIIα neurons on mechanical and thermal pain. (L–N) Effect of chemogenetic activation of CA1 CaMKIIα neurons on cognitive function. *n* = 6 ~ 7 mice per group. *p < 0.05, **p < 0.01, ***p < 0.001 versus Sham + CaMKIIα + mCherry group; ^#^ *p* < 0.05, ^##^ *p* < 0.01, ^###^ *p* < 0.001 versus CCI + CaMKIIα + mCherry group.

In addition to synaptic plasticity, researches have demonstrated that CaMKIIα neuronal activity in hippocampus is integral to the processes of learning and memory [19]. Our findings have indicated a significant reduction in the co‐expression of CaMKIIα and c‐Fos in dCA1 following CCI (Figure 5A,B). To further assess the activity of CaMKIIα^dCA1^ after CCI, we employed fiber photometry to record real‐time calcium transients during FCT (Figure 5C). Notably, during contextual FCT at 28 days post‐CCI, GCaMP activity from CaMKIIα^dCA1^ neurons was significantly diminished (Figure 3D–F), while no substantial differences were observed in tone testing (Figure S2). These results suggest a marked decrease in the activity of CaMKIIα^dCA1^ neurons following CCI. To elucidate the role of CaMKIIα^dCA1^ neurons in CCI‐related behavior, we injected AAV2/9‐CaMKIIα‐hM3D(Gq)‐mCherry into the dCA1 region using stereotactic surgery for chemogenetic activation. Figure 5G illustrates the timeline for viral injection and the subsequent behavioral tests. The viral injection map illustrates the precise location of the injection site (Figure 5H). We found that CaMKIIα^dCA1^ activation did not affect pain hypersensitivity induced by nerve injury (Figure 5I–K), nor did it impact motor performance (Figure S3A,B). However, enhancement of CaMKIIα^dCA1^ neuron activity significantly ameliorated cognitive deficits in CCI mice, as evidenced by reduced latency to reach a hidden platform, increased time spent in the target quadrant in MWM, and prolonged freezing duration during contextual FCT (Figure 5L–N and Figure S3C,D). No significant differences were observed in freezing times during tone tests (Figure S3E,F). These findings suggest that following CCI, not only is synaptic plasticity impaired, but also the activity of CaMKIIα neurons in dCA1 is decreased. Furthermore, enhancing the activity of CaMKIIα^dCA1^ neurons can ameliorate CCI‐induced cognitive impairments without influencing assessments of pain behaviors.

### 3.6 Targeted CHOP Knockdown Ameliorated Cognitive Deficits by Promoting Synaptic Plasticity and Increasing CaMKIIα Neuronal Activity

**FIGURE 6:**
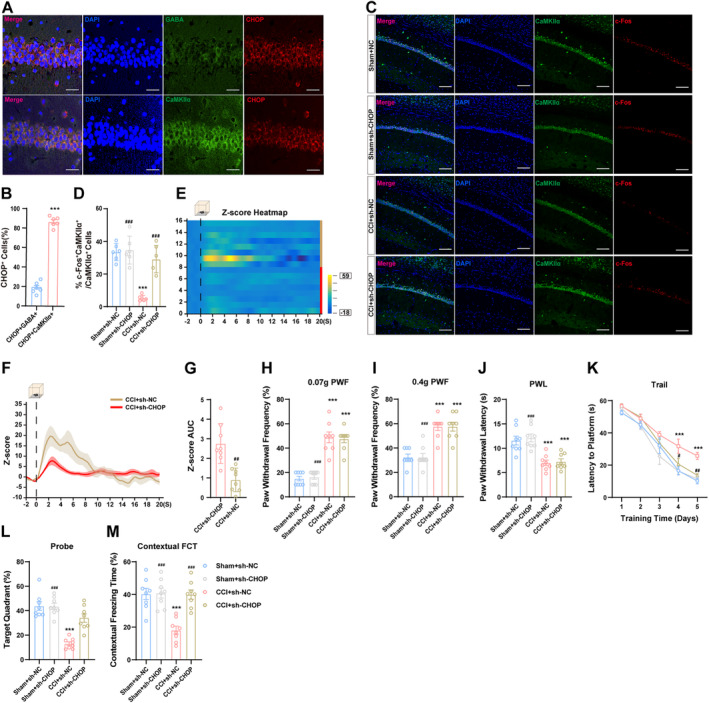
Downregulation of CHOP in CA1 enhances CaMKIIα neuronal activity and alleviates cognitive impairments in CCI mice. (A, B) Representative images of double immunofluorescence for CHOP within the dCA1. Scale bar, 10 μm. (C, D) Effect of CHOP knockdown on the colocalization of c‐Fos/CaMKIIα determined using double immunofluorescence staining. Scale bar, 100 μm. (E–G) Role of CHOP reduction on CA1 neuronal calcium signaling assessed by calcium imaging during FCT context exposure. (H–J) The alteration of mechanical allodynia and thermal hyperalgesia induced by CCI in the presence of CHOP knockdown. (K–M) The outcome of CHOP knockdown on learning and memory deficits triggered by CCI during the MWM and FCT. *n* = 6 ~ 8 mice per group. *p < 0.05, **p < 0.01, ***p < 0.001 versus with the sham + sh‐NC group; ^#^ *p* < 0.05, ^##^ *p* < 0.01, ^###^ *p* < 0.001 versus CCI + sh‐CHOP group.

Our previous findings have confirmed that targeting the knockdown of CHOP can rescue synaptic plasticity impairment following CCI. Given the role of hippocampal neuronal activity in cognitive dysfunction associated with chronic pain, we further investigated whether increased CHOP after CCI affects hippocampal neuronal activity. The immunofluorescence double‐labeling results revealed that CHOP is predominantly expressed in neurons, particularly in CaMKIIα neurons, rather than in microglia or astrocytes (Figures 6A,B and S4). The results depicted in Figure 6C,D show that the decreased expression of CHOP markedly increases the colocalization of c‐Fos and CaMKIIα neurons, indicating a reversal of the reduced activity of CaMKIIα neurons following CCI. Similarly, the reduction in GCaMP activity within CaMKIIα^dCA1^ neurons was reversed following CHOP deletion (Figure 6E–G).

Following an evaluation of CHOP's effects on both synaptic plasticity and activity of CaMKIIα neurons, we conducted further investigations to explore its influence on behavioral phenotypes. Our results suggest that the administration of LV‐shRNA‐DDIT3 did not elicit any alterations in baseline pain thresholds or significantly alleviate CCI‐induced hyperalgesia (Figure S5A,B and Figure 6H–J). Moreover, there were no discernible discrepancies in motor function observed during OFT (Figure S5D,E). Finally, through assessments using MWM and FCT, downregulation of CHOP demonstrated beneficial effects on learning and memory functions; specifically, mice in the CCI + sh‐CHOP group exhibited improved cognitive abilities (Figure 6K–N and Figure S5F–I).

### 3.7 4‐PBA Treatment Reversed the Increase in CHOP Expression, Alleviated Endoplasmic Reticulum Dilation, and Improved Pain and Cognitive Dysfunction Following CCI

**FIGURE 7:**
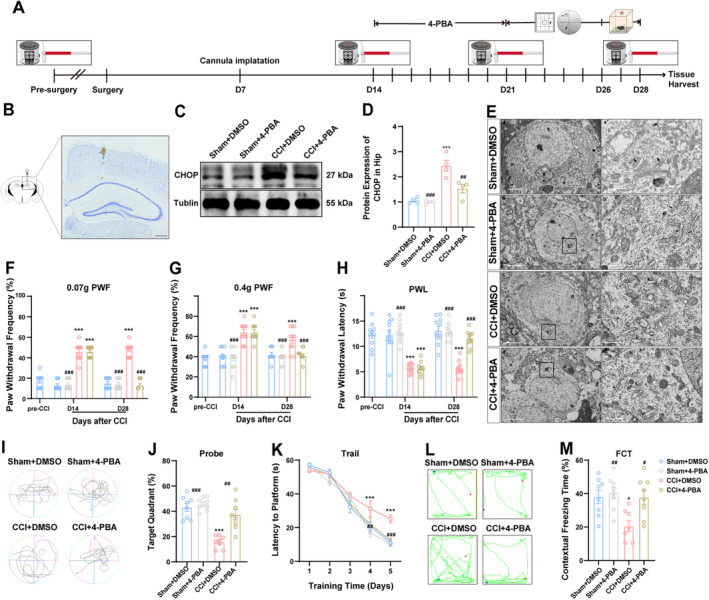
4‐PBA treatment alleviates CCI‐induced pain hypersensitivity and cognitive dysfunction. (A) The time schedule of the experiment. (B) Typical images of the implantation of the cannula by Nissl staining. Scale bar, 500 μm. (C, D) Influence of 4‐PBA treatment on CHOP protein levels in the hippocampus, as revealed by Western blotting. (E) Impact of 4‐PBA treatment on CCI‐triggered endoplasmic reticulum enlargement observed through TEM. The rows indicate the ER. Scale bars, 5 μm (a, b, c, d) and 1 μm (a’, b’, c’, d’). (F–H) Changes in mechanical hypersensitivity and thermal hyperalgesia due to CCI following 4‐PBA treatment. (I–M) Outcomes of 4‐PBA treatment on cognitive impairments caused by CCI. *n* = 4 ~ 10 mice per group. *p < 0.05, **p < 0.01, ***p < 0.001, compared with the sham + DMSO group; ^#^ *p* < 0.05, ^##^ *p* < 0.01, ^###^ *p* < 0.001, compared with the CCI + DMSO group.

Following CCI, the hippocampal ER stress response may undergo significant changes, as indicated by ER dilation and the upregulation of CHOP along with its upstream signaling molecules (Figures 2A, 7E and S1). These alterations suggest that ER stress in the hippocampus may be globally modified, playing a crucial role in neuropathic pain and cognitive impairment following CCI. To elucidate the impact of global intervention on ER stress, this study focuses on applying the broad‐spectrum ER stress inhibitor 4‐PBA. Figure 7A outlines the procedural steps at each time point in this experiment. Nissl staining analysis confirmed precise placement of the catheter in the dCA1 region without inducing any damage to pyramidal layer (Figure 7B). Figure 7C,D show that 4‐PBA effectively reversed the CCI‐induced upregulation of CHOP protein expression, concurrently alleviating the ER expansion triggered by CCI (Figure 7E). Prior to modeling, there were no discernible differences in pain thresholds among the groups (Figure S6A–C). Following the administration of 4‐PBA, there was a significant reduction in allodynia caused by CCI, as depicted in Figure 7F–H. Furthermore, cognitive impairments post‐CCI were also ameliorated with 4‐PBA treatment, as evidenced in Figures 7I–M and S6F–G. No significant impact on exercise capacity was observed (Figure S6D,E). These results suggest that 4‐PBA exerts multifaceted effects on behavioral outcomes.

## 4 Discussion

An expanding corpus of studies involving chronic pain patients and rodent models of chronic pain consistently indicates the presence of cognitive deficits, encompassing a range of cognitive abilities such as learning, memory, attention, and executive function [2, 3, 4]. The interplay between cognitive impairment and chronic pain sensitizes and exacerbates both conditions, further complicating clinical management [5, 20]. However, due to the unclear pathophysiological mechanisms involved and the limited availability of effective therapeutic options in clinical practice, there is an urgent need to explore novel therapeutic targets. Recent reports have implicated ER stress in various neurodegenerative diseases; however, its specific role in chronic pain and cognitive impairment is still not fully understood [10, 11]. In this study, we present the novel finding that elevated expression of CHOP in the hippocampal dCA1 region following nerve injury may play a critical role in chronic pain‐related cognitive impairment by mediating synaptic plasticity and CaMKIIα neuronal activity. Furthermore, specific knockdown of CHOP and administration of the ER stress inhibitor 4‐PBA demonstrate potential therapeutic benefits in alleviating outcomes associated with chronic pain and cognitive impairment.

Chronic pain and its associated comorbidities, encompassing neuropsychiatric disorders such as anxiety and depression, alongside cognitive dysfunction, have garnered significant attention in extensive research endeavors [1, 4]. This study meticulously explores the intricate mechanisms that underpin chronic pain and cognitive deficits. Our observations reveal that cognitive impairment becomes apparent 28 days following nerve injury, whereas no substantial cognitive deficits are detected at 14 days post‐injury. These findings imply that cognitive impairment arising from nerve damage may emerge during the persistent phase of chronic pain rather than during its developmental stage, resonating with a considerable body of existing literature [7, 16]. However, the study conducted by Véronique Morel demonstrated that rats in the SNL model exhibited cognitive impairment 7 days post‐surgery by the Y‐maze test [21]. This finding does not correspond with the timeframe of cognitive impairment reported in certain other chronic pain models [7, 16]. Such discrepancies may arise from variations in animal species, methodologies for assessing cognitive function, and approaches to modeling chronic pain. Furthermore, the observed cognitive impairments cannot be attributed to locomotor dysfunctions, as evidenced by comparable total distances traversed during OFT between groups throughout MWM and FCT assessments.

ER stress is acknowledged as a pivotal factor in the investigation of neurodegenerative diseases [10, 11]. CHOP, a principal effector in ER stress‐induced UPR, plays a crucial role in the onset and progression of various neurodegenerative disorders [12]. In this study, we evaluated the spatial‐temporal expression patterns of CHOP in the ACC, hippocampus, and mPFC following surgical intervention. Given the unique function of the hippocampus in memory and its specific timing of CHOP expression—coupled with evidence of endoplasmic reticulum dilation—our study sought to explore the role of CHOP in neuropathic pain and related cognitive alterations following CCI. However, the potential involvement of CHOP in modulating chronic pain‐related behavioral changes within both ACC and mPFC necessitates further investigation due to their distinct contributions to pain processing and regulation.

Synaptic plasticity represents a fundamental mechanism that underpins cognitive processes [19]. Our study elucidated that nerve damage precipitates a reduction in dendritic spine density within the dCA1 region, while neuronal complexity remains unaltered—a finding that stands in contrast to existing literature suggesting an association between chronic pain and altered neuronal complexity [22]. Considering the dynamic nature of synaptic plasticity, these discrepancies may stem from methodological variations in pain modeling and the timing of brain sampling. Although alterations in dendritic spine morphology are crucial for synaptic plasticity, our investigation was unable to assess spine shape due to the inherent limitations of Golgi staining in accurately quantifying three‐dimensional spine architecture. Moreover, we examined the protein PSD95, which is intricately linked with synaptic plasticity [18]. Our findings reveal a significant decline in PSD95 levels within the hippocampus of mice following CCI, aligning with previous studies. Additionally, we noted changes in LTP at the Schaffer collateral‐CA1 synapse of the mouse hippocampus post‐CCI, thereby affirming the disruptive influence of CCI on hippocampal synaptic plasticity in vitro.

Neuronal activity, in conjunction with neuronal plasticity, plays a pivotal role in the regulation of chronic pain as well as learning and memory processes [5]. Post‐surgical analysis from our study revealed a notable decrease in CaMKIIα^dCA1^ neuronal activity, evidenced by diminished c‐Fos expression and reduced GCaMP signals. The chemical‐genetic activation of CaMKIIα^dCA1^ neurons in CCI mice resulted in cognitive enhancement with minimal effects on pain perception, contrasting sharply with Shuang H's findings where the activation of pyramidal neurons within the dCA1 region via chemogenetics not only mitigated pain perception but also exacerbated short‐term memory deficits observed in the SNI model [23]. Shuang H's research indicated that the analgesic effect from chemogenetically activated CaMKIIα neurons in the dCA1 region is primarily associated with the activation of pyramidal neurons located within layer five of the prelimbic area (PrL), thereby initiating involvement from descending analgesic pathways [23]. These findings suggest that observed variations may be attributed to distinct functions among different nuclei engaged in processing chronic pain and its related behaviors. The hippocampus appears to play a more prominent role in cognitive functions than it does in pain perception [7, 16]. Conversely, both the ACC and mPFC may facilitate mechanisms for pain relief due to their closer association with nociceptive processing [24].

Upregulation of CHOP is recognized as a critical factor driving pathophysiological changes in neurodegenerative diseases, particularly its role in promoting apoptosis [12, 13]. However, TUNEL staining and Western blotting analysis for cleaved caspase‐3 revealed no evidence of apoptosis in this study. Functional analysis demonstrated that CHOP knockdown was associated with improvements in synaptic plasticity, enhanced activity of CaMKIIα neurons, and recovery from learning and memory deficits without affecting pain sensitivity. These results suggest that increased CHOP expression following CCI is not due to neuronal apoptosis but may relate to inhibition of synaptic plasticity and proteins involved in neuronal activity, thereby influencing cognitive deficits observed under chronic pain conditions. CHOP plays a crucial physiological role in recognizing and degrading unfolded proteins within the ER through a proteasome‐dependent pathway and protein–protein interactions [12, 13]. CHOP has been found to interact with FOXO3a, which is involved in synaptic function and regulating neuronal excitability through ion channels [25, 26]. Additionally, PGC‐1α is crucial for mitochondrial function and bioenergetics, and its downregulation could substantially contribute to the onset of pain and cognitive deficits [27]. Chen et al. showed that CHOP binds to PGC‐1α, resulting in reduced levels of PGC‐1α [28]. The aforementioned findings suggest that the function of CHOP needs to be redefined as a regulator of stress responses, rather than being solely considered a pro‐apoptotic factor [13, 25, 28]. Therefore, based on current evidence and literature, elevated CHOP levels post‐CCI are hypothesized to potentially impact cognitive deficits linked to chronic pain by regulating specific intermediary proteins through excessive degradation, which control synaptic plasticity and neuronal excitability.

Our research indicates that after CCI, the UPR may activate various ER stress related pathways to a different extent, leading to the upregulation of CHOP; hence, we employed 4‐PBA, a broad‐spectrum ER stress inhibitor [29]. Our study revealed that 4‐PBA mitigates CCI‐induced ER swelling and increased CHOP expression, while also improving cognitive deficits and enhancing pain tolerance post‐CCI, thus providing a therapeutic effect broader than CHOP knockout. It has been documented in prior research that 4‐PBA can lower the phosphorylation levels of c‐Jun N‐terminal kinase (JNK) and p38, which are essential in pain signaling pathways in the hippocampus [30, 31]. Collectively, unlike the ineffective approach of downregulating CHOP to reduce allodynia after CCI, 4‐PBA treatment effectively increases the pain threshold, potentially through its regulation of various endoplasmic reticulum stress signaling molecules related to chronic pain pathophysiology.

Ultimately, while our study provides novel insights into the mechanisms underlying chronic pain and cognitive impairment, it is important to acknowledge several significant limitations. Firstly, a comprehensive review of the literature and company websites revealed a lack of specific CHOP inhibitors, impeding their clinical application and translational research aimed at reducing CHOP levels to alleviate cognitive deficits associated with chronic pain. Secondly, the mechanisms by which upregulated CHOP influences synaptic plasticity and neuronal activity remain inadequately explored. We propose that CCI‐induced CHOP elevation potentially impairs cognition in chronic pain by degrading proteins crucial for synaptic plasticity and neuronal excitability. Moreover, this study did not provide comprehensive analysis of the relationship between chronic pain and cognitive impairment after CCI, as direct evidence is missing. However, there is evidence suggesting that chronic pain may have several impacts on cognitive function: (a) disruption of brain functions: chronic pain is known to disrupt the brain areas that process pain. This disruption may lead to cognitive impairment by affecting the neural pathways that connect to cognitive processing regions [16, 32]. (b) Memory storage hypothesis: some literature suggests that chronic pain could be a form of memory, potentially occupying cognitive storage space in the brain. This could potentially affect learning and memory processes that are unrelated to pain [20]. (c) Experimental observations: our study found no cognitive dysfunction during the acute phase of pain. However, between 21 and 28 days post‐ligation, which corresponds to the chronic phase of pain, cognitive dysfunction did emerge. Based on the existing literature and our experimental findings, we reasonably infer that chronic pain following CCI could be a significant contributor to cognitive impairment.

## 5 Conclusion

Our findings reveal that peripheral nerve injury can induce ER stress‐related UPR in the hippocampus. Furthermore, the suppression of CHOP following CCI ameliorates chronic pain‐related cognitive dysfunction, which is associated with the restoration of synaptic plasticity and the improvement of CaMKIIα^dCA1^ neuronal activity. These insights deepen our understanding of the pathophysiology underlying neuropathic pain and its associated cognitive deficits, thereby facilitating the development of targeted therapeutic interventions.

## Author Contributions

J.C. W.Z. and S.S. conceptualized the project, secured funding, and oversaw the experimental procedures. Q.M. conducted all animal experiments and electrophysiological recordings, and authored the initial manuscript draft. Y.S. and S.C. executed the Western blotting analysis. X.R., Y.Z. and X.H. established the animal model and conducted immunofluorescence studies. J.D. managed drug administration. L.L. performed the statistical analysis. J.C. Z.Z. and S.S. provided critical revisions to the manuscript concerning its intellectual content. All authors reviewed and approved the final manuscript for publication.

## Ethics Statement

The experimental protocols involving animals were subjected to review and received approval from the Animal Care and Use Committee at Zhengzhou University, adhering strictly to the guidelines established by the National Institutes of Health for the care and use of laboratory animals.

## Consent

The authors have nothing to report.

## Conflicts of Interest

The authors declare no conflicts of interest.

## Supporting information


**Figure S1.** Expression levels of proteins in hippocampus associated with the UPR. (A–C) Western blot analysis of the phosphorylated PERK (p‐PERK) and total PERK protein levels, as well as the phosphorylated IRE1 (p‐IRE1) and total IRE1 protein levels. (D, E) Immunoblotting analysis of ATF6. *n* = 6 mice per group. *p < 0.05, **p < 0.01 and ***p < 0.001 as compared to sham mice.

**Figure S2.** Effect of chemogenetic activation of CaMKIIα^dCA1^ neurons on auditory cue FCT. (A) Illustrative *Z*‐score heatmaps. (B) *Z*‐score AUC for calcium signaling. (C) Statistical analysis of calcium activity. *n* = 8 mice per group. *p < 0.05, **p < 0.01, ***p < 0.001 versus Sham + CaMKIIα + mCherry group; #*p* < 0.05, ##*p* < 0.01, ###*p* < 0.001 versus CCI + CaMKIIα + mCherry group.

**Figure S3.** Behavioral outcomes following chemogenetic activation of dCA1 region CaMKIIα. (A, B) The impact of chemogenetic stimulation on motor performance is assessed using OFT. (C) Track diagram illustrating the performance in the MWM subsequent to chemogenetic activation. (D) Track diagram representing the contextual FCT following chemogenetic activation. (E, F) The effects of chemogenetic activation on performance in the auditory‐cued FCT are depicted. *n* = 7 mice per group. *p < 0.05, **p < 0.01, ***p < 0.001 versus Sham + CaMKIIα + mCherry group; #*p* < 0.05, ##*p* < 0.01, ###*p* < 0.001 versus CCI + CaMKIIα + mCherry group.

**Figure S4.** Representative images of immunohistochemical staining for CHOP localization in the hippocampus. NeuN, IBA1, and GFAP were used as markers for different cell types. Scale bar, 10 μm.

**Figure S5.** Effect of CHOP knockdown on behavioral outcomes. (A–C) The preoperative baseline thresholds for mechanical and thermal pain across the groups. (D, E) Impact of CHOP knockdown on the performance during the OFT. (F) Track diagram of the MWM following CHOP knockout. (G) Schematic representation of the contextual FCT post CHOP knockout. (H, I) Results of CHOP knockdown during the auditory‐cued FCT. *n* = 8 mice per group. *p < 0.05, **p < 0.01, ***p < 0.001 versus with the sham + sh‐NC group; #*p* < 0.05, ##*p* < 0.01, ###*p* < 0.001 versus CCI + sh‐CHOP group.

**Figure S6.** Outcomes of 4‐PBA treatment on behaviors. (A–C) The baseline thresholds for mechanical and thermal pain prior to surgery across all groups. (D, E) Effect of 4‐PBA administration on locomotor activity as measured in OFT. (F, G) Outcomes from 4‐PBA administration in the auditory‐guided FCT. *n* = 8 mice per group. *p < 0.05, **p < 0.01, ***p < 0.001, compared with the sham + DMSO group; ^#^ *p* < 0.05, ^##^ *p* < 0.01, ^###^ *p* < 0.001, compared with the CCI + DMSO group.

## Data Availability

The data and materials supporting the results in this article are available from the corresponding author on reasonable request.
