# Supplementary figures and images for: CHOP‐Mediated Disruption of Hippocampal Synaptic Plasticity and Neuronal Activity Contributes to Chronic Pain‐Related Cognitive Deficits
Source: CNS Neurosci Ther. 2025 Jan 16;31(1):e70160. doi: 10.1111/cns.70160 (PMC11736631; doi:10.1111/cns.70160)

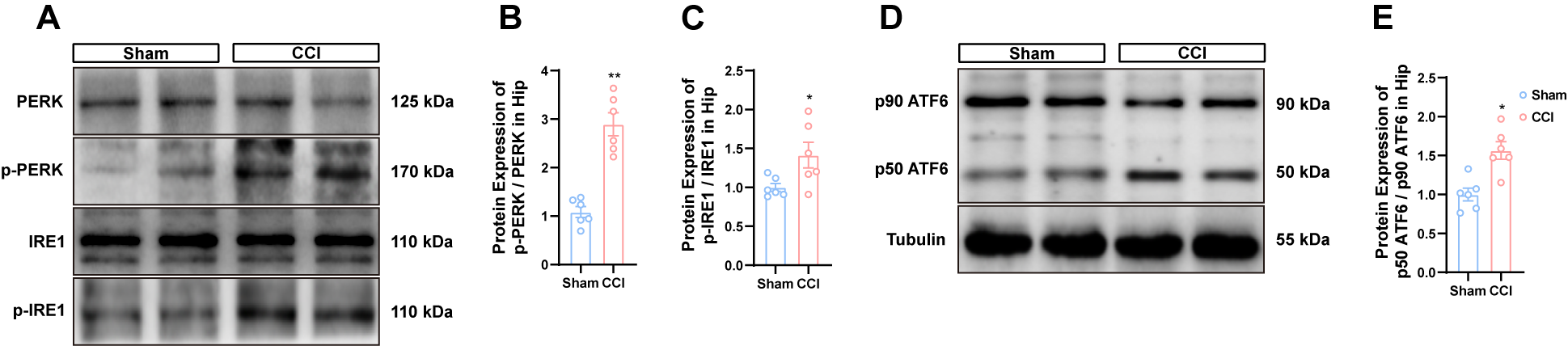

Supplement: Supplementary file 1 — Figure S1. Expression levels of proteins in hippocampus associated with the UPR. (A–C) Western blot analysis of the phosphorylated PERK (p‐PERK) and total PERK protein levels, as well as the phosphorylated IRE1 (p‐IRE1) and total IRE1 protein levels. (D, E) Immunoblotting analysis of ATF6. n = 6 mice per group. *p < 0.05, **p < 0.01 and ***p < 0.001 as compared to sham mice. [file CNS-31-e70160-s002.tif]

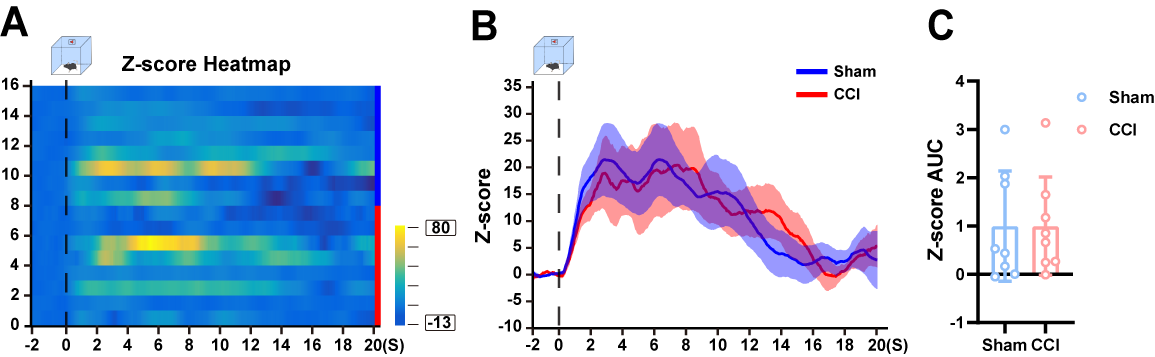

Supplement: Supplementary file 2 — Figure S2. Effect of chemogenetic activation of CaMKIIαdCA1 neurons on auditory cue FCT. (A) Illustrative Z‐score heatmaps. (B) Z‐score AUC for calcium signaling. (C) Statistical analysis of calcium activity. n = 8 mice per group. *p < 0.05, **p < 0.01, ***p < 0.001 versus Sham + CaMKIIα + mCherry group; #p < 0.05, ##p < 0.01, ###p < 0.001 versus CCI + CaMKIIα + mCherry group. [file CNS-31-e70160-s003.tif]

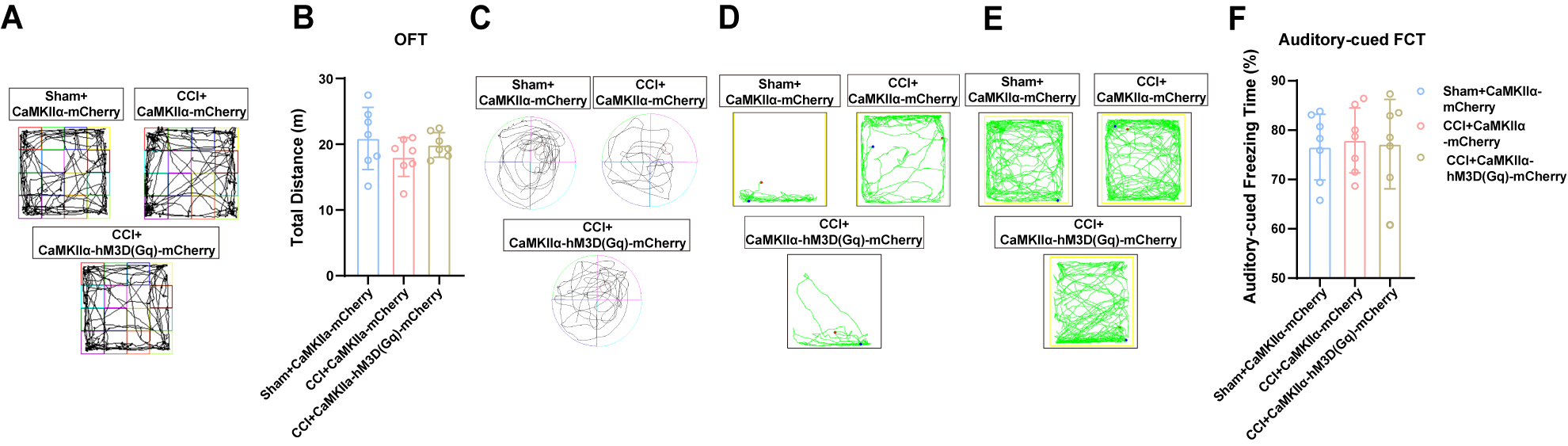

Supplement: Supplementary file 3 — Figure S3. Behavioral outcomes following chemogenetic activation of dCA1 region CaMKIIα. (A, B) The impact of chemogenetic stimulation on motor performance is assessed using OFT. (C) Track diagram illustrating the performance in the MWM subsequent to chemogenetic activation. (D) Track diagram representing the contextual FCT following chemogenetic activation. (E, F) The effects of chemogenetic activation on performance in the auditory‐cued FCT are depicted. n = 7 mice per group. *p < 0.05, **p < 0.01, ***p < 0.001 versus Sham + CaMKIIα + mCherry group; #p < 0.05, ##p < 0.01, ###p < 0.001 versus CCI + CaMKIIα + mCherry group. [file CNS-31-e70160-s005.tif]

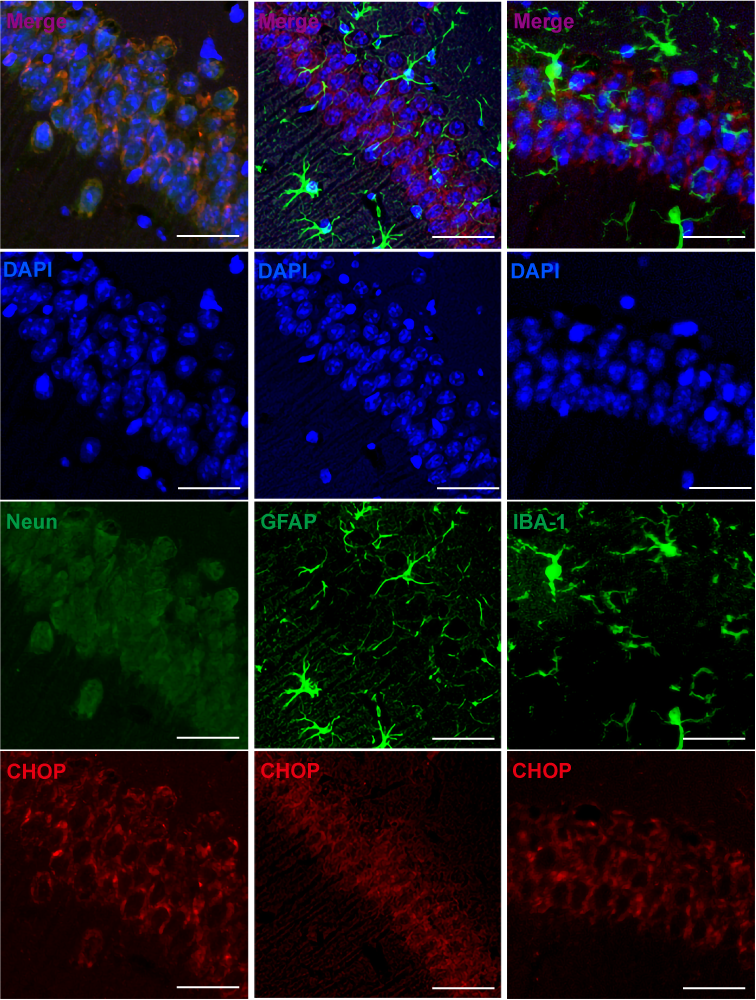

Supplement: Supplementary file 4 — Figure S4. Representative images of immunohistochemical staining for CHOP localization in the hippocampus. NeuN, IBA1, and GFAP were used as markers for different cell types. Scale bar, 10 μm. [file CNS-31-e70160-s004.tif]

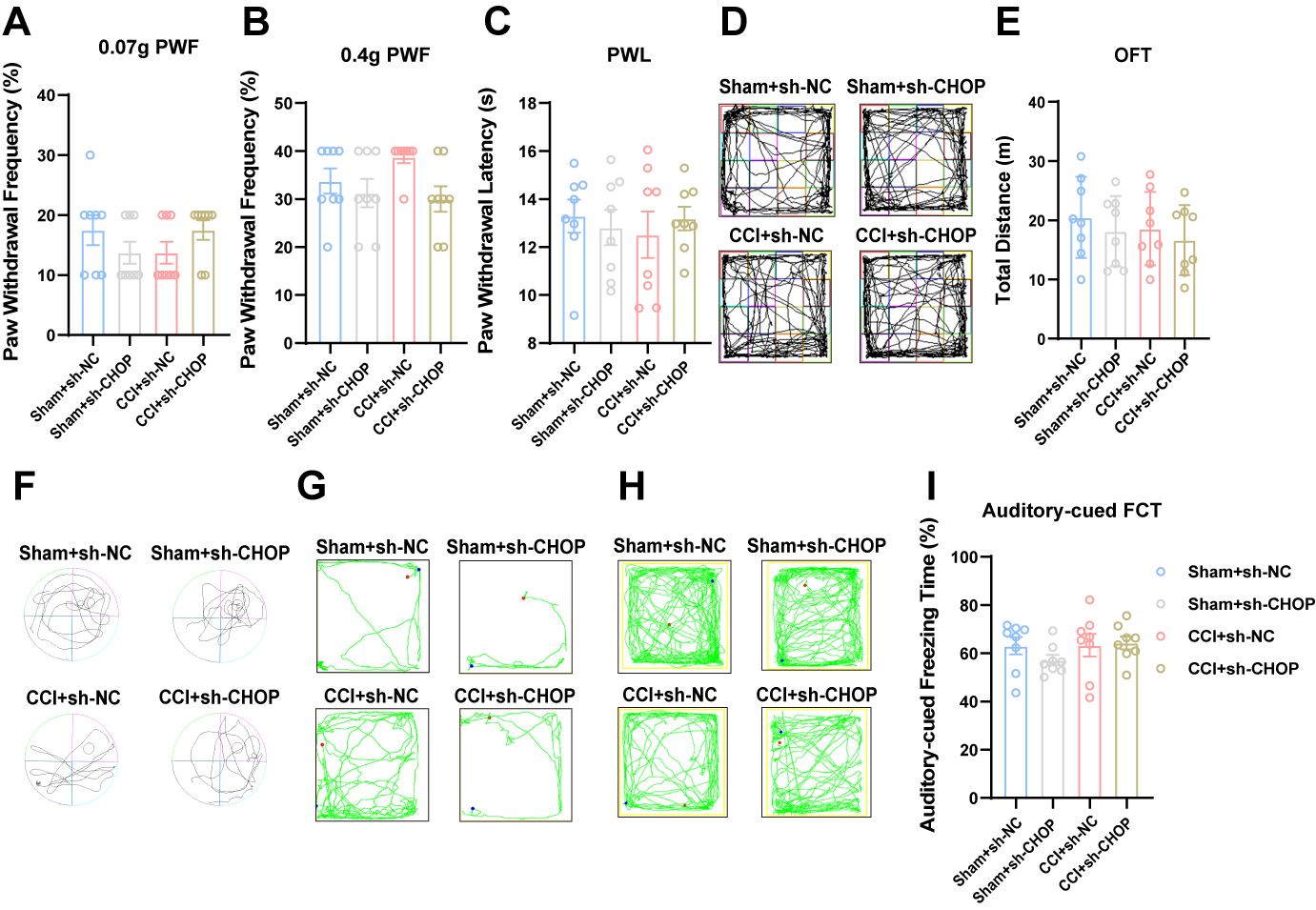

Supplement: Supplementary file 5 — Figure S5. Effect of CHOP knockdown on behavioral outcomes. (A–C) The preoperative baseline thresholds for mechanical and thermal pain across the groups. (D, E) Impact of CHOP knockdown on the performance during the OFT. (F) Track diagram of the MWM following CHOP knockout. (G) Schematic representation of the contextual FCT post CHOP knockout. (H, I) Results of CHOP knockdown during the auditory‐cued FCT. n = 8 mice per group. *p < 0.05, **p < 0.01, ***p < 0.001 versus with the sham + sh‐NC group; #p < 0.05, ##p < 0.01, ###p < 0.001 versus CCI + sh‐CHOP group. [file CNS-31-e70160-s001.tif]

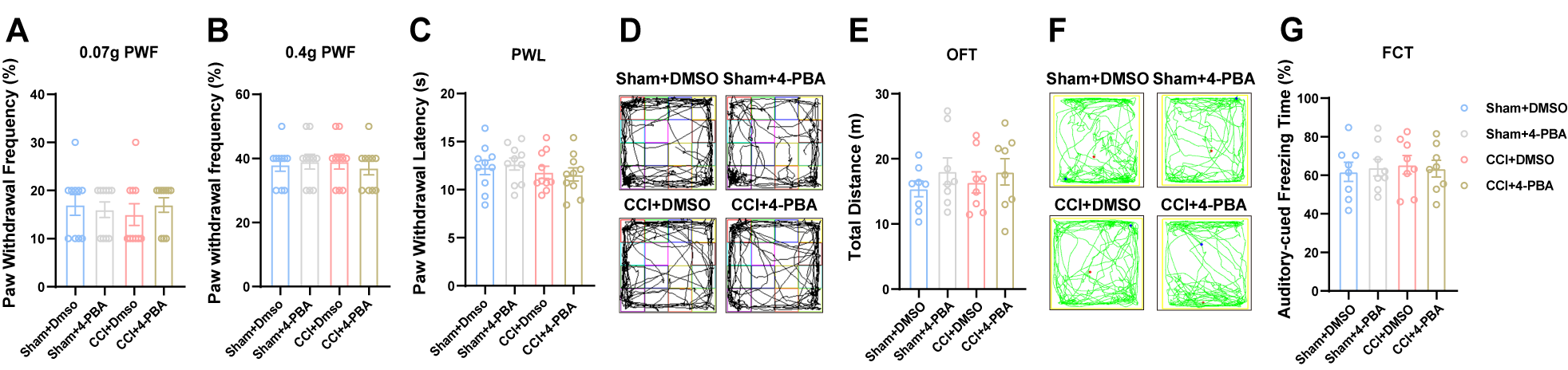

Supplement: Supplementary file 6 — Figure S6. Outcomes of 4‐PBA treatment on behaviors. (A–C) The baseline thresholds for mechanical and thermal pain prior to surgery across all groups. (D, E) Effect of 4‐PBA administration on locomotor activity as measured in OFT. (F, G) Outcomes from 4‐PBA administration in the auditory‐guided FCT. n = 8 mice per group. *p < 0.05, **p < 0.01, ***p < 0.001, compared with the sham + DMSO group; # p < 0.05, ## p < 0.01, ### p < 0.001, compared with the CCI + DMSO group. [file CNS-31-e70160-s006.tif]
